# Identification of tRNA-Related Fragments and Their Potential Regulatory Effects in Thyroid-Associated Ophthalmopathy

**DOI:** 10.3389/fgene.2022.878405

**Published:** 2022-04-04

**Authors:** Zifan Yue, Fei Tong, Chengcheng Zeng, Ruili Wei

**Affiliations:** Department of Ophthalmology, Changzheng Hospital of Navy Military Medical University, Shanghai, China

**Keywords:** thyroid-associated ophthalmopathy, microRNA, tRNA-related fragments, messenger RNA, RNA sequencing

## Abstract

Recently, the potential role of tRNA-related fragments (tRFs) in ophthalmic diseases has been extensively researched. However, systematic studies on the potential regulatory effects of tRFs in thyroid-associated ophthalmopathy (TAO) are lacking. We used high-throughput sequencing techniques to measure expression levels of mRNAs and tRFs in patients with TAO, and the results were verified by real-time quantitative reverse transcription polymerase chain reaction (q-PCR). Next, the potential biological regulatory effect of differentially expressed tRFs was analyzed, and potential downstream target RNAs of differentially expressed tRFs were predicted to explore the potential role of tRFs as therapeutic targets and biomarkers of TAO. A total of 50 tRFs and 361 mRNAs were dysregulated in the TAO group, and tRF5-GluCTC, PMAIP1, HSD17B2 and ATF3 were verified to be significantly differentially expressed in TAO. Our research reveals that several associated pathways likely play a role in the pathogenesis of TAO. By targeting ATF3, HSD17B2 and PMAIP1, tRF5-GluCTC may play a potential role in regulating the orbital fibroblast adipogenic response and fibrotic hyperplasia in patients with TAO.

## Introduction

Thyroid-associated ophthalmopathy (TAO), which is also known as Graves’ ophthalmopathy (GO), is an autoimmune disease that frequently occurs in patients with hyperthyroidism and occasionally in euthyroid or hypothyroid patients (Şahlı and Gündüz, 2017). The most common clinical features of TAO are soft tissue inflammation, eye movement disorders, eyelid retraction, exophthalmos and strabismus. In severe cases, TAO is often accompanied by decreased vision and corneal ulcers, which affect quality of life (Marinò et al., 2020). The pathogenesis of TAO mainly involves autoimmune cross-reactivity of the thyroid and extraocular muscles (Bahn, 2015). Histologically, the orbital fibroblasts (OFs) of TAO patients specifically express TSHR. In TAO, immune tolerance to TSHR, which is specifically recognized by helper T cells and leads to their activation, secreting massive amounts of inflammatory factors and cytokines, is lost; activated helper T cells promote fat formation and hyaluronic acid production and cause connective tissue remodeling, leading to different degrees of orbital fat and extraocular muscle swelling (Smith and Hegedüs, 2017). In addition to TSHR, insulin-like growth factor 1 receptor (IGF-1R) is also highly expressed in the OFs of TAO patients. There is recent strong evidence that IGF-1R participates in TAO pathogenesis. TSHR signal mediation partly depends on functional IGF-1R, and the human IGF-1R inhibitory monoclonal antibody teprotumumab is a breakthrough advancement in TAO treatment (Smith et al., 2017; Douglas et al., 2020). However, the pathogenesis of TAO has not been elucidated in detail, and there are no specific biological indicators for early diagnosis and classification of the severity and activity of TAO. Hence, early diagnosis and effective treatment of active TAO need to be addressed.

Transfer ribonucleic acid (tRNA) is a crucial linker molecule for protein synthesis. It specifically recognizes the codon information of messenger RNA (mRNA) and converts it into the amino acid sequence for polypeptide chain synthesis. Some recent studies have shown that tRNA and its precursors are specifically cleaved into small fragments by specific enzymes (Lee et al., 2009; Anderson and Ivanov, 2014). During this process, the tRNA-derived fragments are of two types: tRNA-related fragments (tRFs) and tRNA halves (tiRNAs) (Anderson and Ivanov, 2014). Second only to miRNAs, tRFs are the second most common small noncoding RNAs (sncRNAs). They are 18–22 nt long and divided into the tRF-5, tRF-3, and tRF-1 series (Lee et al., 2009). tRFs interact with ribosomes and aminoacyl tRNA synthetase to regulate translation (Gebetsberger et al., 2017; Telonis et al., 2019). Furthermore, tRFs inhibit translation in the form of microRNA, participate in the assembly of the precursor rRNA cleavage complex, and directly suppress translation by superseding the translation initiation factor eIF4G, which binds to mRNA (Venkatesh et al., 2016; Gebetsberger et al., 2017; Huang et al., 2017). A large amount of evidence shows that tRFs have an important biological regulatory role in numerous diseases, such as infection, cancer, neurodegeneration and inflammatory and immune conditions (van Es et al., 2011; Blanco et al., 2014; Selitsky et al., 2015; Sharma et al., 2016; Huang et al., 2017). In particular, tRFs play a critical role in human cancer. They can be utilized as therapeutic targets and diagnostic biomarkers in tumor treatment (Lee et al., 2009; Goodarzi et al., 2015; Olvedy et al., 2016; Huang et al., 2017; Zhu et al., 2020). Although tRFs in ophthalmic diseases have been widely researched, including diabetic cataracts and uveal melanoma (Han et al., 2020; Londin et al., 2020), tRF expression and its potential roles in TAO have not yet been analyzed. We speculate that tRFs may be upregulated in TAO patients and are likely to have a regulatory effect on TAO pathogenesis.

We used a high-throughput sequencing technique to examine expression levels of tRFs and mRNAs in TAO patients and verified their expression by qRT-PCR. The biological regulatory effect and clinical significance of target mRNAs of differentially expressed tRFs were then examined. Potential downstream target genes of differentially expressed tRFs were predicted and intersected with differentially expressed mRNAs from sequencing data to explore the potential of tRFs as therapeutic targets and biomarkers of TAO to supply novel clues into TAO pathogenesis and to supply novel treatment strategies for TAO.

## Materials and Methods

### Ethics Approval

Our research was approved by the Ethics Committee of Changzheng Hospital Ethics Committee affiliated with Naval Military Medical University. Implementation of the experiment complied with the Helsinki Declaration. All patients included signed an informed consent form.

### Patient Inclusion and Tissue Sample Acquisition

6 samples of orbital fat/connective tissue were collected from TAO patients after orbital decompression surgery. All TAO patients initially had hyperthyroid exophthalmos, and their thyroid function was in a normal state. The stable period for these patients was greater than 6 months. The patients did not receive corticosteroid pulse therapy or orbital radiation therapy 6 months before the surgery. At the same time, we obtained orbital fat/connective tissues of six healthy control subjects after plastic surgery; thyroid disease, autoimmune and inflammatory disease, or orbital disease history were exclusion criteria for the control group. Age and sex ratios were similar in both the case and control groups.

### RNA Extraction and Sequencing Data Analysis

We used mirVana™ miRNA Isolation Kit (Austin TX, United States) to extract total RNA, which was used to complete tRF sequencing using orbital adipose/connective tissue samples of three TAO patients and three control individuals. RNeasy Micro Kit (QIAGEN, GmBH, Germany) was used to extract RNA used for mRNA sequencing. Then, we qualified the total RNA extracted using an Agilent 2100 Bioanalyzer (Agilent Technologies, Santa Clara, United States) for quality control. We qualified the samples with an RNA integrity number (RIN) ≥ 7 and used them for analysis.

The sample sequencing library was prepared by adding a 3′ end adaptor and a 5′ end adaptor, followed by reverse transcription, RNA signal amplification, cDNA library size selection, and purification. We used an Agilent 2100 Bioanalyzer to perform library quality control and an Illumina NovaSeq 6000 (Illumina, San Diego, CA) to conduct high-throughput RNA sequencing and obtained 150 bp single reads. We used the RNA samples to produce complementary RNA (cRNA) fluorescently labeled for use with the SBC human ceRNA array V1.0 (4 × 180 K, Agilent Technologies, designed by Shanghai Biotechnology Corporation), which included 18,853 mRNA probes, to identify differentially expressed mRNAs. Low Input Quick Amp Labeling Kit, One-Color (Agilent Technologies, Santa Clara, CA, United States) was used to amplify and label total RNA and the RNeasy mini kit (QIAGEN, GmBH, Germany) to purify labeled cRNA. Gene Expression Hybridization Kit (Agilent Technologies, Santa Clara, CA, United States) was employed for hybridization of each slide with 1.65 μg of Cy3-labeled cRNA in a hybridization oven. After 17 h, the slides were washed in staining dishes (Thermo Shandon, Waltham, MA, United States) with Gene Expression Wash Buffer Kit (Agilent Technologies, Santa Clara, CA, United States).

### Data Analysis of tRNA-Related Fragments and mRNAs

We used Fastx to sequence and obtain raw reads and filtered them to obtain clean reads. For the clean reads of each sample, those within 18–40 nt were selected for statistical analysis, and all of the reads were compared with the known tRF database tRFdb (http://genome.bioch.virginia.edu/trfdb/search.php) (Kumar et al., 2015). The base distribution of the first position of tRFs of different lengths and the base distribution statistics of each position of the tRFs were calculated. We used the trimmed mean of M values (TMM) method to normalize the number of reads compared to each tRF and then converted them into transcripts per million (TPM) for standardization of tRF expression; TPM was calculated according to the following formula:

TPM = ${\frac{\text{Number of reads on a TRF}\times 10}{\text{total number of reads }}}^{6}$

We then used edgeR (Robinson et al., 2010) to analyze discrepant tRFs between samples. Differential gene screening conditions were a fold change ≥ 2 and *p* value ≤ 0.05. For mRNAs, we scanned the slides using an Agilent Microarray Scanner (with the default settings). Data were extracted with Feature Extraction software 10.7 (Agilent Technologies, Santa Clara, CA, United States). We normalized the raw data by using R software, and differentially expressed genes were screened using Student’s t test. The selection conditions were the same as those described above.

### Target Gene Prediction and Verification of Differential tRNA-Related Fragments

We used miRanda (http://www.microrna.org/microrna/) (Betel et al., 2008) to predict target genes of the obtained differential tRFs and to identify the intersection of the predicted targeted mRNAs with the differentially expressed mRNAs obtained by sequencing. The numbers of target genes for tRF1-ArgTCG, tRF5-GluCTC and tRF5-GluTTC were 558, 4672 and 5473, respectively.

### Related Bioinformatic Analysis

The ClusterProfiler package in R was applied for related bioinformatic analysis (Gene Ontology functional annotation and Kyoto Encyclopedia of Genes and Genomes pathway enrichment analysis), counting the number of target genes among the biological process, cell component and molecular function categories. Gene Ontology (GO) enrichment analysis was carried out and verified it by Fisher’s exact test, with *p* ≤ 0.05 as the criterion. GO terms that met this criterion were defined as significantly enriched among the differentially expressed mRNAs; the results are shown in a scatter plot. Similarly, the number of tRF target genes that differed in each pathway was counted, and the results are shown in a bar chart.

### QRT-PCR Validation

To validate changes in tRFs detected by RNA-seq, we examined orbital adipose tissue samples after surgery from six TAO patients and six healthy individual samples by quantitative real-time PCR (qRT-PCR). According to the expression levels in the TAO group and the control group (fold-change value (FC) > 2, *p* < 0.05), two upregulated tRFs, namely, tRF5-GluCTC and tRF5-GluTTC, and one downregulated tRF, namely, tRF1-ArgTCG, were chosen for qRT-PCR. Four mRNAs, specifically SORL1, PMAIP1, HSD17B2 and ATF3, were used to verify the results by qRT-PCR. U6 small nuclear RNA (snRNA) and GAPDH were used as controls. We used miScript II RT Kit (QIAGENT) and ReverTra Ace qPCR Kit (TOYOBO, FSQ-101) to reverse-transcribe cDNA RNA and SYBR Green Kit (ABI, 4368708) and miRcute miRNA qPCR Detection Kit (TIANGENT, FP401) to amplify the signal with a QuantStudio 5 Real-Time PCR System (ABI, United States). The reactions were performed following the manufacturer’s standard operating procedures. The primers for mRNAs and tRFs were provided by Shanghai Biotechnology Corporation, and the sequences are listed in Tables 1, 2. PCR was performed in 10-μl reactions.

**TABLE 1:** mRNA primer sequences.

Gene symbol	Forward primer	Reverse primer
SORL1	ACA​CCT​CTT​TGA​AAA​TCC​ACT​GTC​T	AGC​CAT​CTT​TAT​TGC​GCT​CTA​AA
PMAIP1	TCA​GTG​TTG​ATT​TCT​TCG​GTC​ACT	TCT​GAA​CAG​AAG​CAA​TAC​AAT​TTG​AGT
HSD17B2	TCC​AAC​CTG​GAG​GCT​TCC​TA	TCC​AGC​TTT​TCC​CAC​TTG​TCA
ATF3	GCC​GAA​ACA​AGA​AGA​AGG​AGA​A	CAG​CAT​TCA​CAC​TTT​CCA​GCT​T
GAPDH	TGA​CTT​CAA​CAG​CGA​CAC​CCA	CAC​CCT​GTT​GCT​GTA​GCC​AAA

**TABLE 2:** tRF primer sequences.

Genes	Primer sequence (5′ to 3′)
tRF1-ArgTCG	GGA​GGG​AGG​TTA​TGA​TTA​ACT​TTT​A
tRF5-GluCTC	CTG​GTG​GTC​TAG​TGG​TTA​GGA​A
tRF5-GluTTC	TCCCACATGGTCTAGCGG
U6	TTC​GTG​AAG​CGT​TCC​ATA​TTT​T

### Statistical Analysis

Qualitative variables are described by the absolute number and frequency and quantitative variables by the mean and standard deviation. We used the Kolmogorov–Smirnov test to examine all variables for a normal distribution. Two-tailed Student’s t tests were employed to compare discrepancy between the two groups, with *p* ≤ 0.05 indicating statistical significance. SPSS Statistics version 26.0 (IBM/SPSS, Inc., IL, United States) was used for all statistical analyses.

## Results

### Differential Expression of tRNA-Related Fragments and mRNAs in the Paired Thyroid-Associated Ophthalmopathy and Normal Groups

**FIGURE 1:**
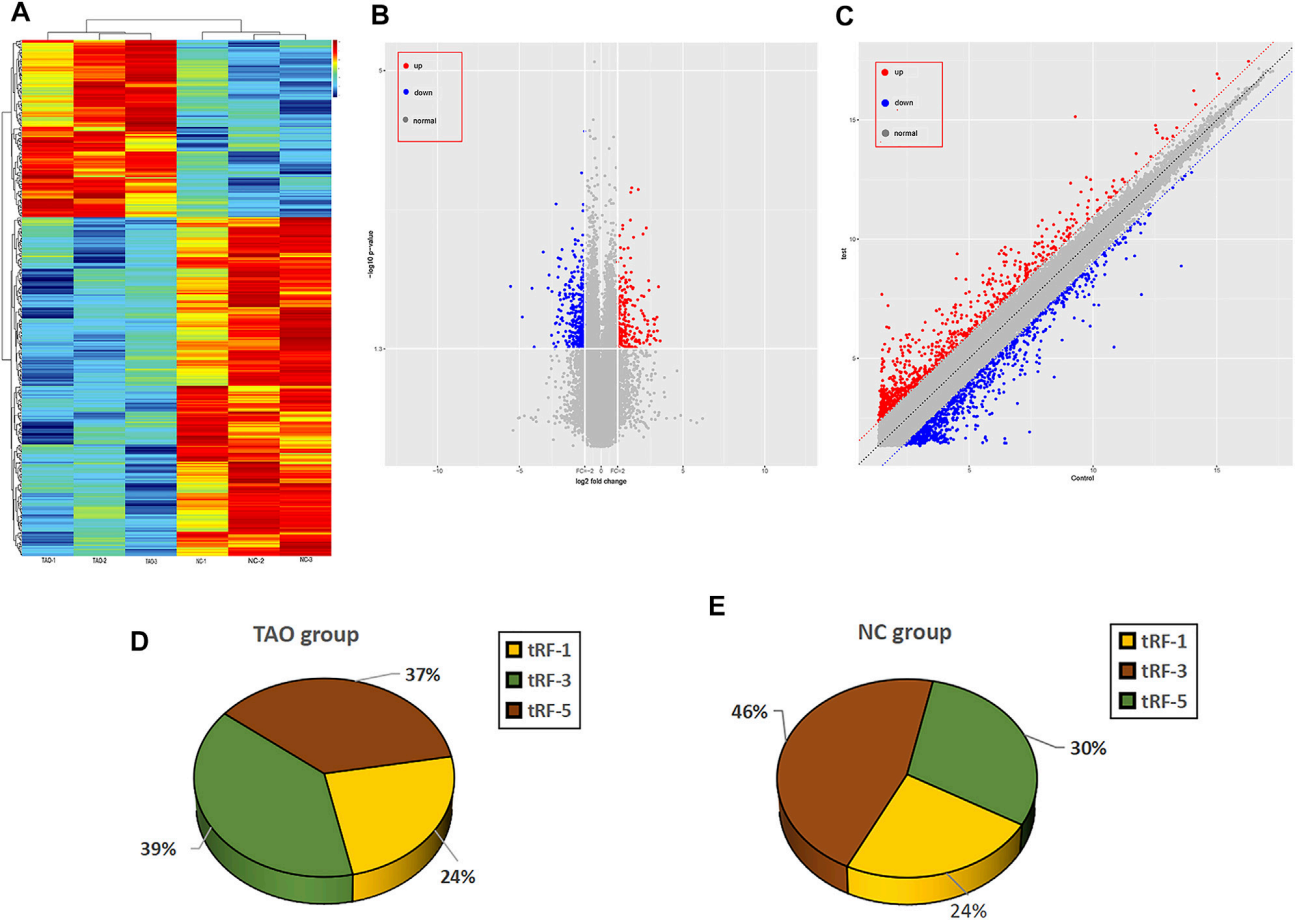
Overview of the expression profiles of mRNA and tRNA-related fragment sequencing data in paired TAO patients versus the normal control group. **(A)** Hierarchical clustering heatmap analysis of the mRNA sequencing data of the paired TAO group and the normal group. The abscissa of the main image is the sample name, and the samples are sorted (grouped) according to the calculated distance; the ordinate is the gene symbol. **(B)** The volcano plot presents differential expression between the groups. Red dots indicate upregulated genes, blue dots indicate downregulated genes, and gray dots indicate genes with no significant differences. **(C)** Scatter plot between the two groups. Red dots indicate upregulated genes, and blue dots indicate downregulated genes; gray dots represent genes with no significant differences. **(D)** Pie charts for all tRFs of each group using all uniquely expressed tRFs. The expression level percentage of each subtype of tRF in the TAO group is shown in the pie chart. **(E)** The expression level percentage of each subtype of tRF in the control group is shown in the pie chart.

The raw and processed sequencing data can be found at GEO (https://www.ncbi.nlm.nih.gov/geo/query/acc.cgi?acc=GSE186480). A total of 50 tRFs were differentially expressed in the TAO group, which could be find in Supplementary Table S1, with 3 tRFs significantly differentially expressed under the criteria of FC > 2 and *p* < 0.05, of which 1 tRF was downregulated and two upregulated. In addition, 361 mRNAs were dysregulated in the TAO group. According to hierarchical clustering analysis, TAO samples could be distinguished from normal samples based on expression of differentially expressed mRNAs (Figure 1A), and volcano plots and scatter plots were generated to visualize differential mRNA expression of between the TAO group and normal group (Figures 1B,C).

### Subtype Distribution of tRNA-Related Fragments Expressed in the Thyroid-Associated Ophthalmopathy and Normal Groups

**FIGURE 2:**
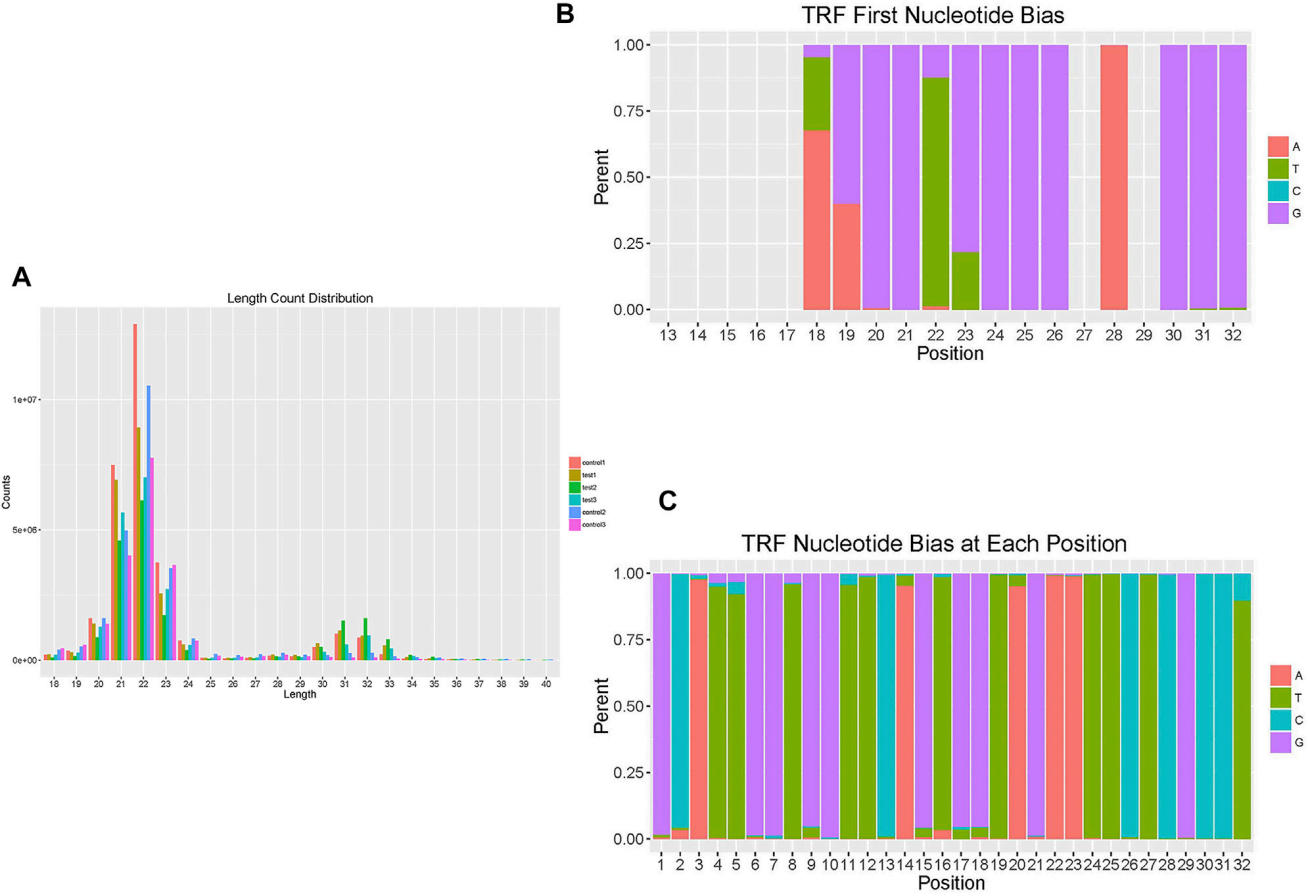
Overview of the read length and position distribution of tRFs in the TAO group and the normal group. **(A)** The bar chart shows the read counts and read length in each sample to illustrate the read length distribution. **(B)** The bar chart shows the base distribution of the first position of tRFs of different lengths. The abscissa is the length of the tRFs, and the ordinate is the percentage of A/U/C/G as the first base. **(C)** The bar chart shows the base distribution of each position of tRFs. The abscissa is the base position of tRFs, and the ordinate is the percentage of the bases A/U/C/G at that position.

As mentioned above, 50 tRFs were differentially expressed between the TAO group and normal group, though not all tRFs met the *p* < 0.05 criterion. The tRFs were sorted into subtypes based on site and length. The tRF subtypes expressed in the two groups are depicted in a pie chart in Figures 1D,E; expression levels of tRF-1 and tRF-3 were reduced but that of tRF-5 increased in the TAO group. The read counts and read lengths in each sample are shown in a bar chart of the read length distribution (Figure 2A). As there might be a strong bias for cleavage at the first base of a tRF accurately conveys the intended meaning.Therefore, we compared all reads against tRFdb and calculated the base distribution of the first position of tRFs of different lengths as well as the base distribution statistics of each tRF position (Figures 2B,C).

### Association of tRNA-Related Fragments Target Genes With Differentially Expressed mRNAs

To explore the underlying regulatory effect of tRFs in TAO pathogenesis, we identified associated target genes for the three validated tRFs (tRF1-ArgTCG, tRF5-GluCTC and tRF5-GluTTC) based on miRanda algorithms. The numbers of target genes for tRF1-ArgTCG, tRF5-GluCTC and tRF5-GluTTC were 558, 4672 and 5473, respectively. After determining the intersection of the differentially expressed mRNAs obtained by sequencing, only 16 mRNAs remained (Table 3).

**TABLE 3:** Intersecting mRNAs between differentially expressed mRNAs obtained by sequencing and predicted target genes of tRFs.

Gene symbol	Target tRF ID	Up/down
SORL1	tRF5-GluCTC	Up
PMAIP1	tRF5-GluCTC	Up
HSD17B2	tRF5-GluCTC	Up
ATF3	tRF5-GluCTC	Up
TRIM9	tRF5-GluCTC	Up
VASH1	tRF5-GluCTC	Up
FRZB	tRF5-GluCTC	Up
CCDC170	tRF5-GluTTC	Up
SLC8A1	tRF5-GluTTC	Up
BMF	tRF5-GluTTC	Up
ZNF667	tRF5-GluTTC	Up
MARCO	tRF5-GluTTC	Up
MEX3B	tRF5-GluTTC	Up
VASH1	tRF5-GluTTC	Up
LPAR1	tRF5-GluTTC	Up
DPP4	tRF5-GluTTC	Up

### Bioinformatic Analyses

**FIGURE 3:**
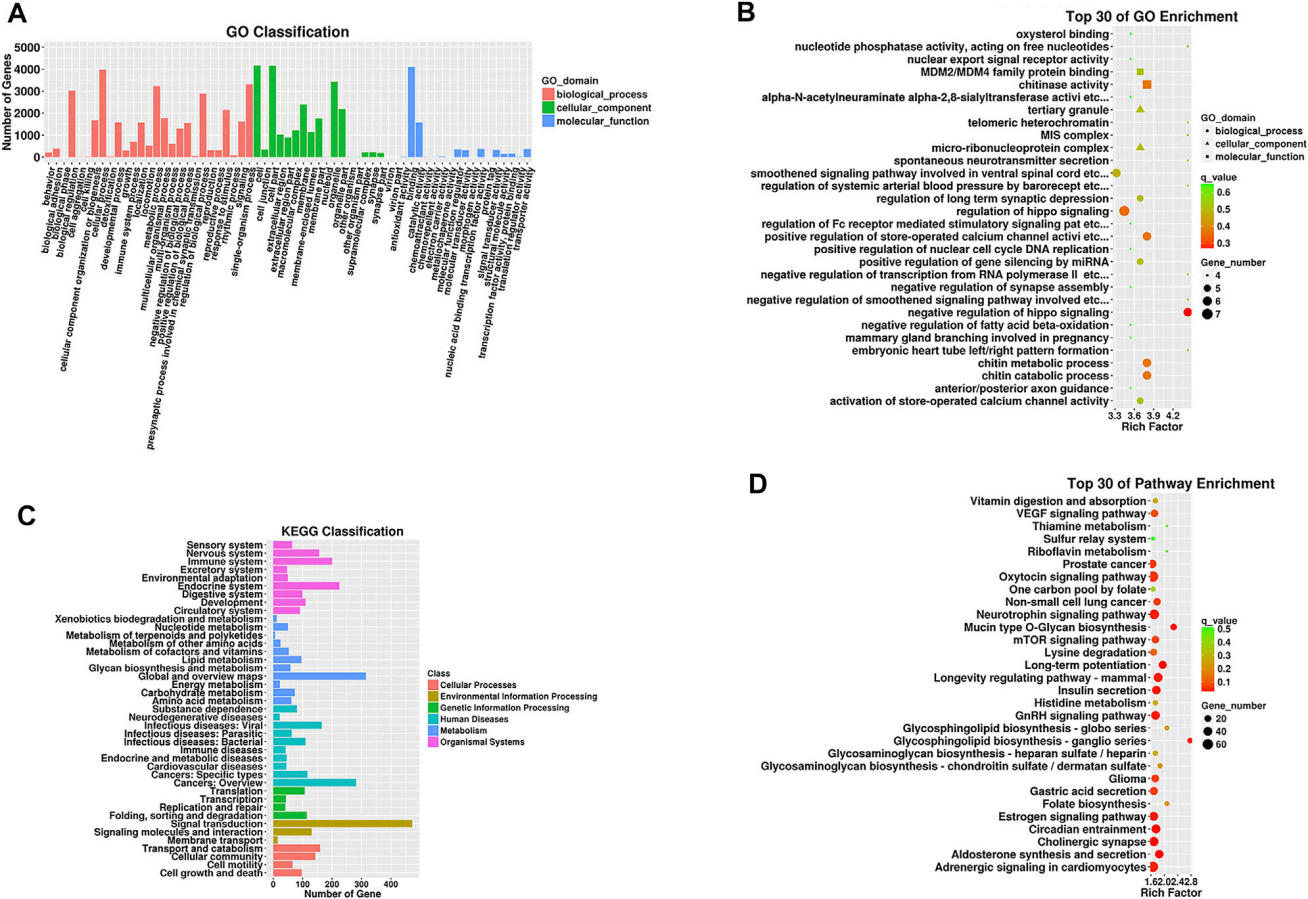
GO functional and KEGG pathway enrichment analyses for target genes of tRFs differentially expressed in the TAO group. **(A)** The GO classification of predicted target mRNAs. **(B)** The KEGG classification of predicted target mRNAs. **(C)** The top 30 GO terms in biological process (BP), cellular component (CC) and molecular function (MF) categories for predicted target genes. **(D)** The top 30 enriched KEGG pathways for the predicted target genes. Enrichment Factor = (number of tRF target genes in a GO term or pathway/number of all target genes in the GO or KEGG database)/(number of genes contained in a GO term or pathway/total number of genes in the database).

We performed functional enrichment analysis of 3 significantly expressed tRFs to evaluate underlying biological functions and corresponding pathways and unveil potential regulatory genes. The results are displayed in Figure 3 and reflect that the biological processes (BPs) of the target genes are mainly related to metabolic processes, biological regulation and cellular component organization. Regarding the cellular component (CC) category, cell parts and membrane parts were notable. For molecular function (MF), the target genes are related to protein binding, antioxidant activity, transcription factor interactions and translation-regulated effects. According to KEGG pathway enrichment analysis, the majority of the potential target genes are involved in the mTOR signaling pathway, VEGF signaling pathway and glycosaminoglycan biosynthesis. The specific results are shown in Supplementary Tables S2 and S3.

### Validation of Differential Expression Levels of tRNA-Related Fragments and mRNAs

**FIGURE 4:**
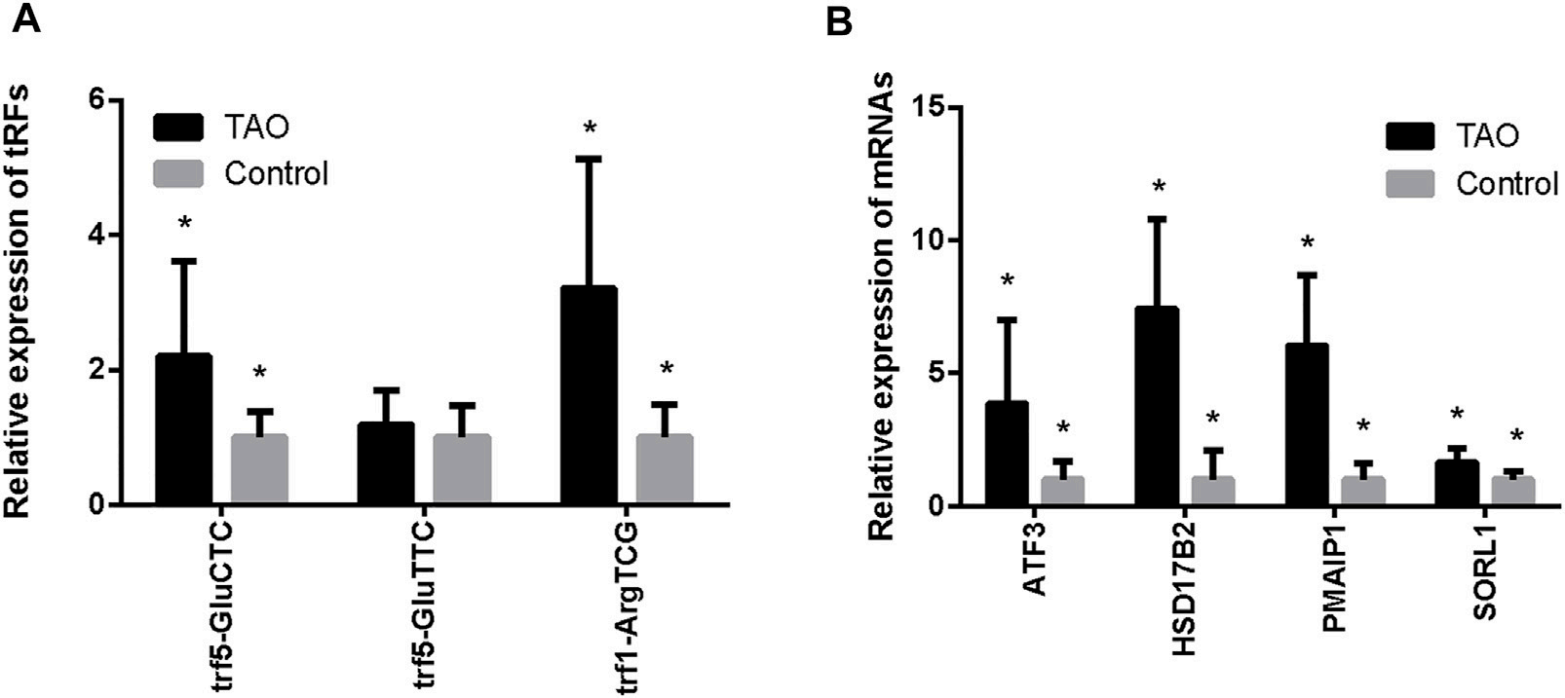
Validation of expression levels of tRFs and intersecting mRNAs in the TAO group and the normal group. **(A)** Expression levels of tRFs detected by q-PCR. **(B)** Expression levels of mRNAs detected by q-PCR (*p < 0.05).

We performed q-PCR to confirm expression changes in the two upregulated and one downregulated tRF. Differential expression of 4 chosen mRNAs (SORL1, PMAIP1, HSD17B2, and ATF3) was also verified. The q-PCR results revealed that tRF5-GluCTC was significantly overexpressed in the TAO group, as confirmed by sequencing; PMAIP1, HSD17B2 and ATF3 were also more highly expressed in the TAO group (Figure 4). The specific results are shown in Supplementary Tables S4 and S5.

## Discussion

The pathogenesis of TAO is a complex biological process that has always puzzled clinicians and scientists and has not been elucidated thus far. What is certain is that it is a self-reactive lymphocyte-induced autoimmune disease in which immune tolerance is lost, leading to an immune response to TSHR that initiates remodeling of the orbital tissue. Recently, many researchers have tried to further explore TAO pathogenesis by comparing differentially expressed RNAs and proteins between control groups and case groups(Yue et al., 2021). In our research, among 50 tRFs found to be dysregulated in the TAO group by RNA sequencing, 2 tRFs (tRF5-GluCTC and tRF5-GluTTC) were upregulated and one (tRF1-ArgTCG) downregulated. Based on target gene prediction, several target genes are highly associated with TAO pathogenesis, including IGF-1R, IL-16 and IL-17A, which are associated with tRF5-GluTTC (Khong et al., 2016; Smith and Janssen, 2019; Xu et al., 2020), CD34, which is associated with tRF5-GluCTC (Wu et al., 2016), and IL-6R, which is associated with tRF1-ArgTCG (Chen et al., 2005). IGF-1R is expressed at high levels in the OFs of TAO patients, and TSHR signal mediation partly depends on functional IGF-1R. IGF-1R stimulation leads to fat formation, hyaluronic acid synthesis, chemokine production, IL-16 release and regulatory activation, T cell expression, and promotion of lymphocyte transport into the orbit. There is much progress in research on the human IGF-1R inhibitory monoclonal antibody teprotumumab in TAO treatment (Khong et al., 2016; Smith and Janssen, 2019). Studies have found that CD34-expressing fibrocytes are remarkably increased in TAO and invade orbital tissues, where they become CD34 ^+^ fibroblasts and are thus included in the population of OFs. These CD34 ^+^ fibroblasts also express TSHR and produce inflammatory response-induced inflammatory cytokines; they differentiate into adipocytes or myofibroblasts, causing tissue reconstruction in TAO patients. However, these genes, which correlate highly with the pathogenesis of TAO, were not differentially expressed in our TAO group, as verified by mRNA sequencing, perhaps because their effect does not occur at the transcriptional level. Furthermore, GO analysis revealed that the potential role of tRFs in TAO mainly involves biological regulation and translation regulator activity, which may be because tRFs can inhibit mRNA translation, similar to miRNAs, or can competitively suppress binding of elongation factor eIF4G to mRNA, decreasing protein translation (Sobala and Hutvagner, 2013; Huang et al., 2017). In addition, pathway enrichment analysis for target genes showed that most potential target genes participate in the mTOR and VEGF pathways. mTOR signaling is indispensable in mammalian biological regulation and is related to many cell survival processes, such as autophagy, preservation of cellular metabolic balance and energy generation (Mizushima et al., 2008). mTOR signaling also has a vital effect on TAO pathogenesis. Wang et al. (2019) reported that steroidogenic factor 1 affects TAO development through mTOR signaling, and Kim et al. (2020) found that insulin-like growth factor-binding protein (IGFBP)-secreting placenta-derived mesenchymal stem cells (PD-MSCs) downregulate the mTOR pathway to inhibit adipogenesis in OFs from TAO patients. VEGF has a central role in the pathogenesis of diverse cancers and blinding eye diseases (Apte et al., 2019), and serum VEGF levels are significantly increased in patients with active TAO, reflecting the degree of ocular inflammatory activity and long-standing autoimmune processes in orbital and thyroid tissues. Increased VEGF levels also promote thyroid gland angiogenesis (Kajdaniuk et al., 2014; Ye et al., 2014). However, the relationship between the above 3 tRFs and mTOR/VEGF signaling needs further exploration.

Our PCR results showed that tRF5-GluCTC is highly dysregulated in TAO patients and probably plays a regulatory role through the verified differentially expressed target mRNAs ATF3, HSD17B2 and PMAIP1. ATF3 is a transcription factor induced by stress with a crucial function in regulating tumorigenesis, metabolism and immunity. ATF3 is expressed at a low level in normal or quiescent cells; its presence inhibits differentiation of adipocytes, whereas its absence leads to increased fat accumulation in macrophages (Ku and Cheng, 2020). Additionally, ATF3 plays a regulatory role in the production of myofibroblasts and IL-6 (Müller et al., 2017; Soraya et al., 2021), which have a significant effect on the pathogenesis of TAO. We also found that HSD17B2 acts as a lipid biosynthesis-associated marker and probably has a regulatory effect on orbital fat production (Lee et al., 2015), and PMAIP1 plays a regulatory role in the proliferation of fibroblasts through p53 pathway (Nagpal et al., 2016; Okuda et al., 2017). Therefore, we speculate that tRF5-GluCTC acts in the pathogenesis of TAO by targeting ATF3, HSD17B2 and PMAIP1. In TAO, overexpressed tRF5-GluCTC may regulate ATF3, HSD17B2 and PMAIP1 mRNA levels to modulate the adipogenic response and proliferation of OFs, adjusting adipogenesis and fibrosis in the orbit. Nevertheless, the exact role of tRF5-GluCTC and overexpression of the 3 mRNAs in the pathogenesis of TAO and whether there is a clear targeting effect between tRF5-GluCTC and these 3 mRNAs remain unclear.

There are several limitations of this research. First, OFs from TAO patients at the active stage were not included. The first-line treatment for active TAO is methylprednisolone pulse therapy, which is often used before orbital decompression surgery. Because RNA expression is probably influenced by dexamethasone, it is difficult to acquire OFs from patients with active TAO who have not received methylprednisolone pulse therapy. Second, as the number of cases used for sequencing was small, the representativeness was not ideal, and although the sequencing results were verified, the sequencing method we used did not include all tRNA-derived fragment types. Third, there is no recognized animal model of TAO, and we plan to perform animal experiments in our future research. Despite these limitations, our research is an important attempt to explore the potential relationship between tRFs and the pathogenesis of TAO.

In conclusion, we examined 50 differentially expressed tRFs and 361 differentially expressed mRNAs in orbital adipose tissue between TAO patients and healthy individuals, and bioinformatic analysis suggested that differentially expressed tRFs have a regulatory effect on mTOR/VEGF signaling, and based on qRT-PCR verification, we speculate that tRF5-GluCTC may play a potential role in regulating OFs in the adipogenic response and fibrotic hyperplasia of TAO patients by targeting ATF3, HSD17B2 and PMAIP1. Putative genes and signaling pathways were revealed, and a novel perspective for future studies on the exact mechanism of TAO is provided. The findings provide innovative ideas for further research on TAO biomarkers and potential therapeutic targets.

## Data Availability

The datasets presented in this study can be found in online repositories. The names of the repository/repositories and accession number(s) can be found below: https://www.ncbi.nlm.nih.gov/, GSE186480.

## Abbreviations

OFs, orbital fibroblasts; tRFs, tRNA-related fragments (tRFs); TAO, thyroid-associated ophthalmopathy; tiRNAs, tRNA halves.
